# Study on the Anti-Adenovirus Mechanism of *Sargassum fusiforme*

**DOI:** 10.3389/fcimb.2022.860559

**Published:** 2022-03-07

**Authors:** Guanrong Feng, Duo Zhang, Chengcheng Peng, Mingjiang Wu, Pengpeng Xiao, Nan Li

**Affiliations:** ^1^ Institute of Virology, Wenzhou University, Wenzhou, China; ^2^ Key Laboratory of Virology and Immunology of Wenzhou, Wenzhou University, Wenzhou, China; ^3^ College of Life and Environmental Science, Wenzhou University, Wenzhou, China

**Keywords:** human adenovirus, *Sargassum fusiforme*, antiviral activity, endoplasmic reticulum stress, *in vitro*

## Abstract

Human adenovirus (HAdV) has a worldwide distribution and remains a major pathogen that leads to infections of the respiratory tract. No specific treatments or vaccines are yet available for HAdV infection. *Sargassum fusiforme*, an edible seaweed, has attracted a lot of attention for its various bioactivities. *S. fusiforme* has been reported to exhibit antiviral activity. However, research studies about its anti-HAdV activity are few. In this research, we found that *S. fusiforme* had low cytotoxicity and possessed anti-human adenovirus type 7 (HAdV7) activity *in vitro*, and the most effective ingredient was alginate. The time of addition assay demonstrated inhibitory effects that were observed in all life stages of the virus. In addition, we observed that the antiviral activity of alginate against HAdV7 infection might be closely related to the endoplasmic reticulum stress (ERS) pathway. Taken together, these results suggest that *S. fusiforme* extracts have potential application in the prevention and treatment of HAdV infection.

## Introduction

Human adenovirus (HAdV) belongs to the Adenoviridae mammalian adenovirus family (genus *Mastadenovirus*). It is a linear double-stranded DNA virus without an envelope (Rowe et al., 1953). The core of HAdV is composed of four structural proteins (PV, PVII, TP, and PX) and DNA together, with PVII being the core protein that wraps DNA. Presently, more than 85 HAdV types have been identified, which were divided into seven species (A–G) (Lu et al., 2020). Of these, about 47 types can cause acute infectious zoonoses (Ismail et al., 2019). Related literature illustrated that there were differences in the types of HAdV circulating in different countries or regions and in the same region at different times (Lynch et al., 2018; Fu et al., 2019). Acute respiratory disease induced by HADV-7 accounted for more than 60% of the global incidence rate in Asia and posed a threat to public health (Xu et al., 2018). So far, there are no effective drugs against HAdV. Commercial adenovirus vaccine is presently available only in the United States (Majee et al., 2020).

*Sargassum fusiforme* is a large seaweed belonging to the Sargassaceae family, Fucales order, and Phaeophyceae class (Liu et al., 2020b). It grows chiefly in coastal areas of Asian countries, such as Japan, South Korea, and North Korea (Liu et al., 2020a). There are abundant proteins, polysaccharides, and microelements in *S. fusiforme*, which has a wide range of biological activity in terms of antioxidant (Li Y. T. et al., 2018), antitumor (Fan et al., 2017), immunity (Sugiura et al., 2016), anti-aging (Bogie et al., 2019), bone growth and blood glucose (Zhou et al., 2021), anticoagulant, growth, and development. Moreover, Paskaleva et al. showed that *S. fusiforme* extract inhibited more than 90% of HIV type 1 (HIV-1) infection and replication in T cells, human macrophages, and microglia and more than 70% of pseudotyped HIV-1 [vesicular stomatitis virus (VSV)/NL4-3] infection in human astrocytes (Paskaleva et al., 2006). A separate study suggested that there exist at least two different bioactive molecules in *S. fusiforme* extract: one inhibiting HIV-1 fusion by interacting with the CD4 receptor and the other directly inhibiting the HIV-1 reverse transcriptase (Paskaleva et al., 2008).

In this study, we showed that *S. fusiforme* extracts had potential antiviral HADV-7 ability and that the most effective ingredient was alginate. At the same time, we compared three different types of anti-adenoviruses, and the stage of interaction between alginate and adenovirus was the most effective.

## Materials and Methods

### Cells, Viruses, and Reagents

A549 cells were generously offered by Dr. Yiquan Li, Changchun University of Chinese Medicine. A549 cells were cultured in Dulbecco’s modified Eagle’s medium (DMEM) supplemented with 10% fetal bovine serum (FBS) and maintained in a humidified 5% CO_2_ incubator at 37°C. Cells infected with HAdV7 were maintained in DMEM supplemented with 2% FBS. HAdV7 was preserved in our laboratory. *S. fusiforme* was kindly provided by Professor Mingjiang Wu, College of Life and Environmental Sciences, Wenzhou University. *S. fusiforme* was picked from a Dongtou algae farm in Wenzhou city, Zhejiang Province. The seaweeds were dried and then powdered. *S. fusiforme* polysaccharides (SFPSs) were extracted by boiling and soaking in diluted HCl. After boiling, 4 M CaCl_2_ was added to the filtrate for precipitation and the supernatant was precipitated with ethanol. The concentration was then freeze dried to obtain *S. fusiforme* fucoidan (Zhang Y. et al., 2020). After soaking in diluted HCl, NaOH was added to the filtrate to maintain the pH at 7.0–8.0, ethanol was added for concentration, and then the sediment was freeze dried to obtain the alginate.

### Cell Viability Assay

The Cell Counting Kit-8 (CCK8) assay kit was purchased from Solarbio (Beijing, China). In brief, A549 cells were cultured in 96-well plates for 12 h and subsequently treated with 10 μl of *S. fusiforme* extracts (powder, fucoidan, and alginate) at different concentrations. Each extract was diluted in six parallel duplicate wells and cultured in humidified 5% CO_2_ at 37°C for 72 h. The cell viability of A549 cells was detected using the corresponding kit in strict accordance with the kit’s instructions.

### Agarose Gel Electrophoresis

After infection of A549 cells, viral DNA (hexon) was extracted using the Biospin Virus DNA/RNA Extraction Kit (BioFlux, Hangzhou, China). PCR was completed with 2×Pfu PCR Mix (TIANGEN, Beijing, China) using the following primers:

Forward primer: 5′-GCCGCAGTGGTCTTACATGCACATC-3′

Reverse primer: 5′-CAGCACGCCGCGGATAAAGT-3′

Electrophoresis was performed using PCR products of 287-bp-long segments with 2% (*v*/*v*) agarose gel.

### Relative qPCR of Viral DNA

The cell supernatant was processed using a Biospin virus DNA/RNA Extraction Kit (BioFlux, Hangzhou, China), followed by measurement of the DNA (hexon) levels in QuantStudio 3 using Top Green qPCR Supermix (Trans, Beijing, China). The PCR conditions comprised 42°C for 20 min; 95°C for 10 min; and 40 cycles of 95°C for 10 s, 58°C for 15 s, and 72°C for 20 s, with a final extension at 72°C for 5 min. The primers used to detect HAdV7 (hexon) and GAPDH (internal control) were as follows:

HAdV7 forward primer: 5′-TGAAATAGCCATAGGCAACAATC-3′

HAdV7 reverse primer: 5′-CGGGCAGAGTAATGTTAGTTGG-3′

GAPDH forward primer: 5′-AAGGTCATCCCTGAGCTGAA-3′

GAPDH reverse primer: 5′-TGACAAAGTGGTCGTTGAGG-3′

Data were analyzed using the 2^−ΔΔCt^ method, and all PCR reactions were performed in triplicate.

### TCID_50_ Assay

Plastic 96-well plates were seeded with A549 cells and incubated at 37°C. After adherence, monolayer A549 cells were infected with a 10-fold serially diluted virus solution, each group with eight parallels. The number of cytopathic pores at each dilution was observed and recorded, until there were no new lesions. Finally, the median tissue culture infective dose (TCID_50_) of virus tissue cells was calculated according to the Reed–Muench method.

### Time of Addition Assay

To examine the anti-HAdV7 effect of alginate in different stages of replication, the addition time of alginate (50 μg/ml) and HAdV7 was split into four groups: pretreatment (2 h before HAdV7 infection), co-treatment (during HAdV7 infection), posttreatment (2 h after HAdV7 infection), and full treatment (2 h before HAdV7 infection to the end of the experiment) (**Figure 4A**). The cell proliferation rate and antiviral activity were examined after 72 h.

### Western Blot

Cells cultured in 10-cm dishes were harvested in RIPA lysis buffer added with a mixture of phosphatase and protease inhibitor (Cwbiotech, Beijing, China). The cell lysates were separated by 10% SDS-PAGE, and the proteins were transferred into a nitrocellulose membrane. After blocking with 5% skimmed milk for 2 h, the membrane was incubated with an anti-Bip antibody (cat. #3177s), anti-PERK antibody (cat. #3192), anti-p-PERK antibody (cat. #3179s), anti-eIf2α antibody (cat. #5324), anti-p-eIF2α antibody (cat. #3398S), and anti-IRE1α antibody (cat. #3294) (all from Cell Signaling Technology, Danvers, MA, USA), an anti-ATF4 antibody (sc-390063), anti-hexon antibody (lot #F2817), and an anti-ATF-6α antibody (sc-166659) (all from Santa Cruz Biotechnology, Santa Cruz, CA, USA) overnight under 4°C. Then, it was incubated with secondary antibodies for 1 h at room temperature. The signals were detected with an enhanced chemiluminescence (ECL) system (ECL, Solarbio, Beijing, China) reaction.

### Statistical Analyses

Statistical analysis was performed by applying *t*-test on the data between two groups. Statistical significance was considered when *p < 0.05, **p < 0.01, and ***p < 0.001.

## Results

### Cytotoxicity of *Sargassum fusiforme* Extracts on A549 Cells

**Figure 1:**
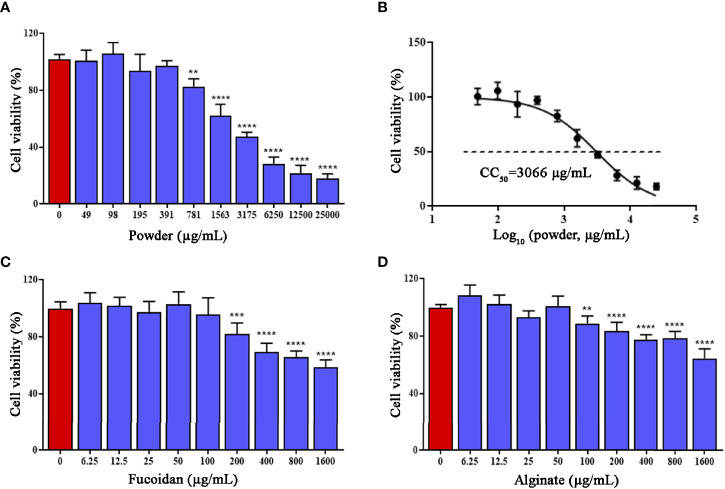
Cytotoxicity of *Sargassum fusiforme* extracts in A549 cells. **(A)** Powder. **(C)** Fucoidan. **(D)** Alginate. A549 cells were treated with *S. fusiforme* extracts at the indicated concentrations at 37°C. The cell viability was detected using the CCK-8 assay. **(B)** The 50% cytotoxic concentration (CC_50_) of powder. **p < 0.01, ***p < 0.001, and ****p < 0.0001.

Prior to examining the antiviral effects against HAdV7, we performed the CCK-8 assay to determine the proper concentration(s) of *S. fusiforme* extracts with minimal cytotoxicity on A549. The detected concentration range was 0–25,000 µg/ml. As shown in **Figure 1A**, the effect of *S. fusiforme* powder under 400 µg/ml treatment on the cell viability was not evident, while the effect of a higher concentration was significant. The 50% cytotoxic concentration (CC_50_) of the powder was 3,066 µg/ml (**Figure 1B**). Moreover, a marked effect on the cell viability was observed with fucoidan and alginate concentrations above 100 and 50 µg/ml, respectively (**Figures 1C, D**). Therefore, concentrations of *S. fusiforme* powder below 400 µg/ml, fucoidan below 100 µg/ml, and alginate below 50 µg/ml were selected for subsequent studies to prevent drug toxicity.

### *Sargassum fusiforme* Extracts Presented Anti-HAdV7 Ability in A549 Cells

**Figure 2:**
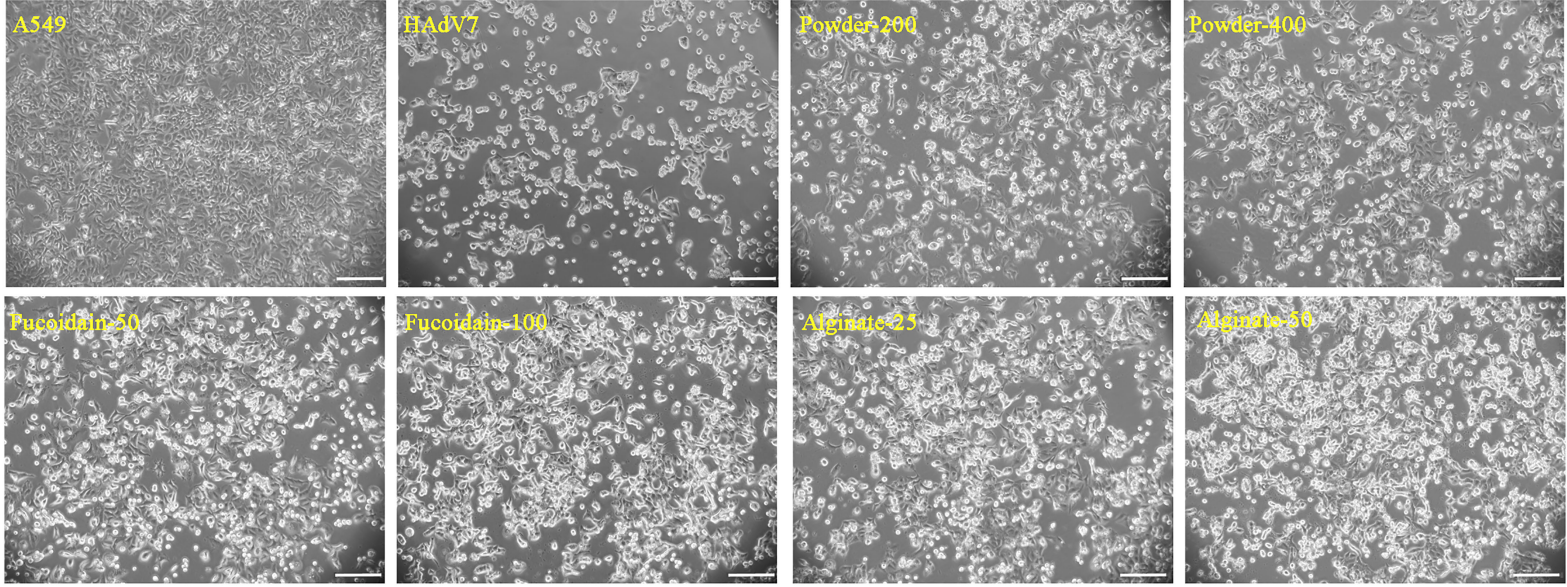
*Sargassum fusiforme* extracts reduced the human adenovirus type 7 (HAdV7)-induced cytopathic effect (CPE) in A549 cells. *Scale bars*, 200 µm.

To determine the anti-HAdV7 activity of *S. fusiforme* extracts, A549 cells were infected with HAdV7 under the indicated concentrations of *S. fusiforme* extracts for 72 h. Subsequently, the cytopathic effect (CPE) of the infected cells was observed. Our results revealed that the mock group cells showed morphological integrity, while the cells in the HAdV7-infected group were shrunken, rounded, and detached and the diacaustic rate was enhanced (**Figure 2**). The CPE in HAdV7-infected cells was remarkably reduced after treatment with *S. fusiforme* extracts, especially in the alginate group.

**Figure 3:**
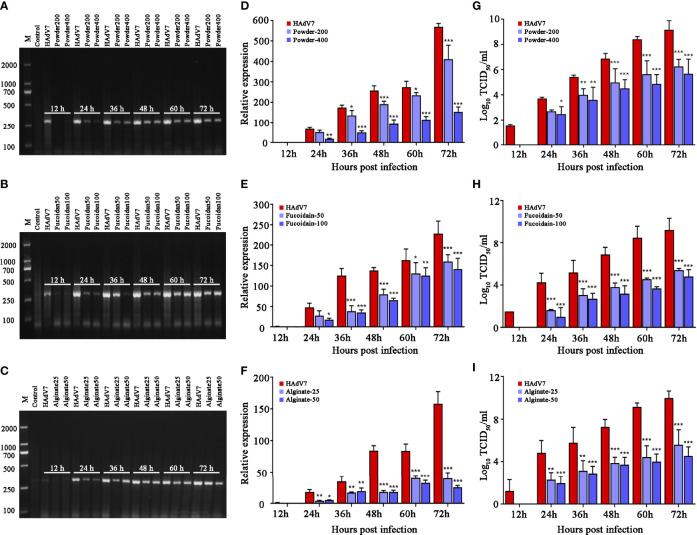
Antiviral activity of *Sargassum fusiforme* extracts on human adenovirus type 7 (HAdV7) infection in A549 cells. **(A–C)** Agarose gel electrophoresis of DNA amplification (hexon) in A549 cells treated with different concentrations of *S. fusiforme* extracts. **(D–F)** Relative quantitative PCR (qPCR) analysis of the virus DNA level. **(G–I)** Analysis of the median tissue culture infective dose (TCID_50_) of viral titers. *p < 0.05, **p < 0.01, and ***p < 0.001.

The intracellular viral DNA, amplified products, and virus progeny in the supernatants were respectively detected using agarose gel electrophoresis, relative quantitative PCR (qPCR), and TCID_50_ assays (**Figure 3**). The results implied that the *S. fusiforme* extracts decreased viral production and the progeny virus yield, with the alginate group showing the best effect. Therefore, we focused on alginate and further explored its effect on HAdV7.

### *Sargassum fusiforme* Extracts Inhibited HAdV7 in Both Early and Late Stages of Infection

**Figure 4:**
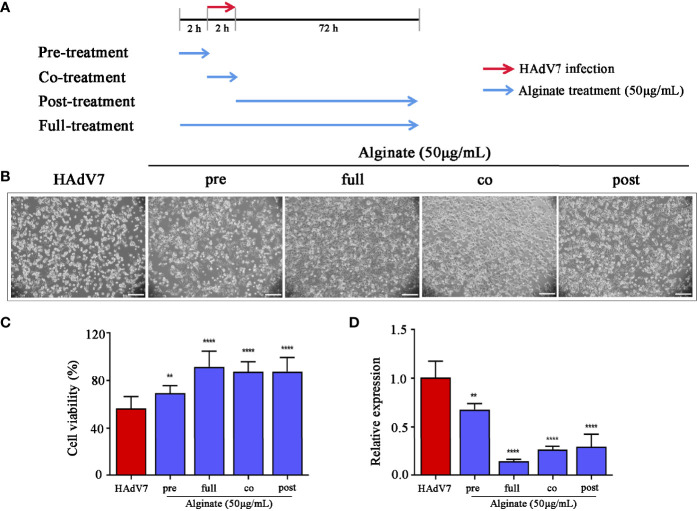
Inhibitory effect of *Sargassum fusiforme* extracts on the life cycle of the virus in A549 cells. **(A)** Experimental scheme of the time of addition assay. **(B)** Human adenovirus type 7 (HAdV7)-induced cytopathic effect (CPE) on A549 cells with the addition of *S. fusiforme* extracts at different time points. *Scale bar*, 200 µm. **(C)** Cell viability was detected using the CCK-8 assay. **(D)** The virus DNA level was detected using relative quantitative PCR (qPCR). **p < 0.01 and ****p < 0.0001.

To pinpoint at which stage of the life cycle of the virus do the *S. fusiforme* extracts work, a time of addition assay was performed in accordance with the scheme (**Figure 4A**). The HAdV7 group was set as a negative control and the full-treatment group as a positive control. CPE was observed under a microscope after 72 h treatment. The results showed that HAdV7-caused CPE were clearly reduced under the full-treatment and co-treatment conditions (**Figure 4B**). Subsequently, the cell viability, as well as the levels of HAdV7 DNA in cells, was determined. The results showed that alginate had the strongest inhibitory effect in the co-treatment stage, indicating that the compound may exert its inhibitory effect mainly during the early stage of HAdV7 infection. In addition, partial effects were observed under the posttreatment stage, suggesting that the compound may also have impact during the late stage of infection with HAdV7 (**Figures 4C, D**).

### *Sargassum fusiforme* Extracts Inhibited HAdV7 Infection *via* the PERK and ATF6 Pathways

**Figure 5:**
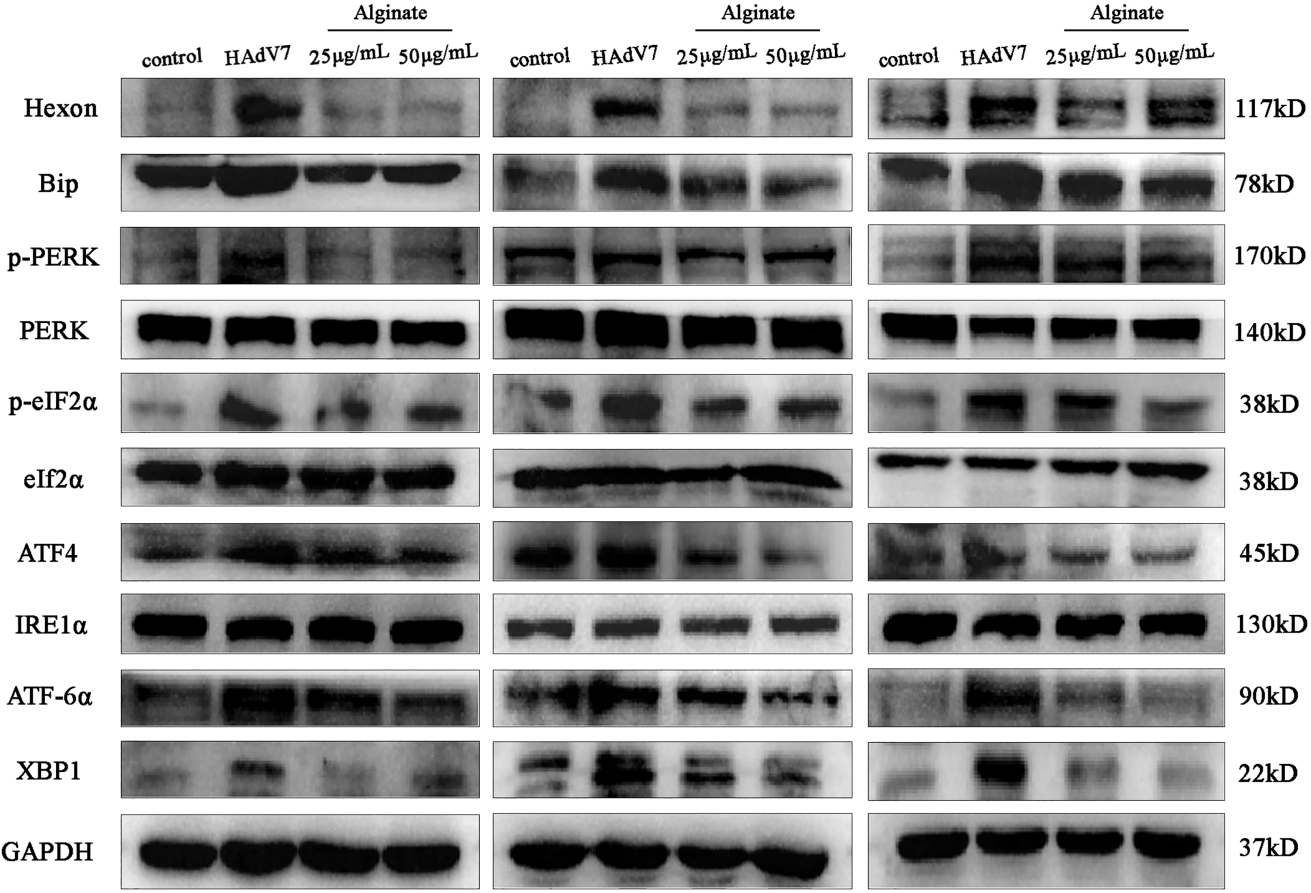
*Sargassum fusiforme* extracts inhibited the endoplasmic reticulum stress (ERS) pathway activated by human adenovirus type 7 (HAdV7) infection.

To elucidate the mechanism of *S. fusiforme* against HAdV7 infection, we focused on the endoplasmic reticulum stress (ERS) and unfolded protein response (UPR) pathways. Bip is a sensitive marker of ERS. Therefore, we first examined the changes of the protein expression of Bip in A549 cells. The results indicated that HAdV7 infection upregulated the expression of the Bip protein, while it was reduced by alginate treatment (**Figure 5**). We then detected which branch of the three UPR signaling pathways (PERK, ATF6, and IRE1) was activated. Our results showed that the levels of p-PERK, p-eIF2α, ATF4, ATF6α, and XBP1 were obviously increased in HAdV7-infected cells, whereas the levels of total PERK, eIF2α, and IRE1α were unaltered. Moreover, the increased expressions of p-PERK, p-eIF2α, ATF4, and ATF6α, which were enhanced by HAdV7 infection, could be reduced with alginate treatment. These results indicate that the suppressive effect of alginate on HAdV7 infection depended in part on the activation of the PERK and ATF6 pathways, but not the IRE1 pathway.

## Discussion

HAdV is widely distributed and is a major cause of respiratory infections in humans. HAdV infection is often self-limiting; however, for children with low immunity and those in special populations, the infection may cause more serious consequences (Li J. et al., 2018; Bautista-Gogel et al., 2020). There are no specific drugs or vaccines available to control HAdV infection; the focus of clinical treatments of adenovirus infections is mostly to reduce the symptoms of patients (Liu et al., 2018; Tian et al., 2018). However, inadequate efficacy and numerous adverse reactions have been observed with such treatments in many cases (Lynch and Kajon, 2021). Consequently, discovering new and effective anti-HAdV agents is particularly urgent. Herein, we demonstrated, for the first time, the antiviral effect of *S. fusiforme* against HAdV7 infection.

*S. fusiforme* is a nutrient-rich edible brown alga that has attracted much attention due to its various biological activities, being widely used in the field of functional food (Zhang R. et al., 2020). Moreover, it has also exhibited antiviral activity (Sun et al., 2021). A previous study demonstrated that treatment of HEp2 cells with *S. fusiforme* extracts in non-cytotoxic concentrations could led to an obvious decrease of respiratory syncytial virus (RSV) replication, RSV gene transcription, RSV protein synthesis, RSV-induced cell death, and syncytium formation (Chathuranga et al., 2021). Sun et al. also reported that, upon treatment with *S. fusiforme* extracts, the gene and protein expressions of the avian leukosis virus subgroup J (ALV-J) were clearly reduced (Sun et al., 2019). Thus, the anti-HAdV7 effect of the *S. fusiforme* extracts was discussed in the present study.

Excessive concentrations of medicinal herbs could lead to cytotoxicity and complicate clinical trials (Palasamudram Shekar et al., 2019). Therefore, we firstly isolated three *S. fusiforme* extracts and then determined their cytotoxic effects on A549 cells. The concentrations of the *S. fusiforme* extracts used in this anti-HAdV7 study were no more than 400 µg/ml (powder), 100 µg/ml (fucoidan), and 50 µg/ml (alginate) in order to prevent drug-induced cytotoxicity and maintain A549 cells showing over 90% vitality.

Thereafter, the anti-HAdV7 effect under different concentrations of *S. fusiforme* extracts was assessed. The results suggested that treatment with *S. fusiforme* extracts suppressed the viral DNA production, amplified products, and progeny yield. Moreover, the results implied that alginate showed the best effect and was therefore chosen in the subsequent study. The time of addition experiment was performed in order to justify at what stage of the HAdV7 life cycle did alginate work. The results suggested that alginate exerted its anti-HAdV7 activity in all life stages of the virus. Noticeably, the inhibition of the intracellular viral DNA level and cell viability was the strongest at the co-treatment stage.

The endoplasmic reticulum (ER) is an essential organelle in eukaryotes that is mainly responsible for protein assembly, modification, folding, calcium storage, and lipid synthesis (Metcalf et al., 2020). Studies have shown that when viruses interact with cells, large amounts of viral proteins accumulate in the ER lumen, which perturbs the homeostasis and function of the ER, triggering the UPR (Prasad and Greber, 2021; Shaban et al., 2021; Shi et al., 2021). Regulation of the ERS on UPR signals is mainly dependent on three pathways (ATF6, PERK, and IRE1). A previous study demonstrated that the porcine reproductive and respiratory syndrome virus (PRRSV) targets the UPR master regulator Bip for degradation and activates both the XBP1s and ATF4 signaling branches at the early stage of infection (Gao et al., 2019). Subsequent studies showed that adenovirus infection also enhanced the UPR (Prasad et al., 2020). Therefore, we wondered whether *S. fusiforme* extracts could inhibit HAdV7 *via* the UPR pathway. The Western blot analysis data revealed that alginate inhibited the Bip level in HAdV7-infected A549 cells. Furthermore, HAdV7-infected cells activated the PERK and ATF6 pathways, but not the IRE1 pathway. This effect also occurred in hepatitis C virus (HCV)- or encephalomyocarditis virus (EMCV)-infected cells (Hou et al., 2014; Wang et al., 2014), indicating that the PERK and ATF6 pathways play a major role in the infection process of multiple viruses. However treatment with alginate blocked the activation of the PERK and ATF6 pathways. Possibly, it can be speculated that *S. fusiforme* may effectively inhibit HAdV7 infection by altering the activation of the UPR pathway.

In conclusion, our study demonstrated that *S. fusiforme* had low cytotoxicity and possessed anti-HAdV7 activity *in vitro*, and the antiviral effects were likely associated with the ERS pathway. Our current study was limited to the application of *S. fusiforme* in HAdV7 infection *in vitro*. Therefore, it is also necessary to establish a virus-sensitive animal model for *in vivo* experiments and to clarify its potential mechanism so as to provide a theoretical basis to guide the clinical application of antiviral drugs.

## Data Availability Statement

The original contributions presented in the study are included in the article/supplementary material. Further inquiries can be directed to the corresponding authors.

## Author Contributions

Conceptualization: NL, PX, and GF. Data curation: GF and NL. Funding acquisition: NL and PX. Investigation: GF, CP, DZ, MW, and PX. Validation: GF and PX. Writing—original draft: GF. Writing—review and editing: NL and PX. All authors have read and agreed to the published version of the manuscript.

## Funding

This study was supported by the Natural Science Foundation of Zhejiang Province (LQ21H160001), the National Natural Science Foundation of China (32002312), and Science and Technology Project of Wenzhou, Zhejiang, China (Y20210080 and Y2020103).

## Conflict of Interest

The research was conducted in the absence of any commercial or financial relationships that could be construed as a potential conflict of interest.

## Publisher’s Note

All claims expressed in this article are solely those of the authors and do not necessarily represent those of their affiliated organizations, or those of the publisher, the editors and the reviewers. Any product that may be evaluated in this article, or claim that may be made by its manufacturer, is not guaranteed or endorsed by the publisher.
